# Timely, Cheap, or Risk-Free? The Effect of Regulation on the Price and Availability of New Drugs

**DOI:** 10.3390/pharmacy12020050

**Published:** 2024-03-18

**Authors:** Laura Levaggi, Rosella Levaggi

**Affiliations:** 1 Faculty of Engineering, Free University of Bolzano-Bozen, Piazza Università, 1, 39100 Bolzano, Italy; laura.levaggi@unibz.it; 2 Department of Economics and Management, University of Brescia, Via San Faustino 74b, 25100 Brescia, Italy

**Keywords:** regulation, personalised drugs, value-based prices, performance-based prices, welfare analysis

## Abstract

The high level of regulation of innovative drugs on the market, which is necessary to protect consumers, produces important effects on drug availability and innovation. In public healthcare systems, the need to curb prices comes from expenditure considerations. The aim of price regulation is to obtain a more equitable allocation of the value of an innovative drug between industries and patients (by reducing prices to make drugs more affordable), but it may also reduce access. (In the listing process, the industry may find it more convenient to limit commercialisation to profitable subgroups of patients.) Furthermore, with the advent of personalised medicine, there is another important dimension that has to be considered, namely, incentives to invest in drug personalisation. In this paper, we review and discuss the impact of different pricing rules on the expenditure and availability of new drugs.

## 1. Introduction

The market for drugs is highly regulated in order to ensure consumer protection. Furthermore, the high cost of some of the active principles makes them unaffordable for most patients to the point that their cost is usually financed either by private insurance or by the public healthcare system. Even in the USA, where private insurance and copayment are the predominant financing methods, prices for new drugs have become an extremely important issue on the political agenda [1,2,3] because they may be responsible for the increase in healthcare bills. In Europe, where drug expenditure is predominantly financed through a public healthcare system, the need to curb expenditure has required stringent regulation, whose effect on the market has been rather controversial [4,5,6,7,8,9,10]. Pricing policies across countries are heterogeneous, ranging from direct pricing (as in Italy and France) to indirect regulation (as in Germany and Japan) and profit control [11,12,13,14]. Also, countries often use more than one method at the same time, leading to pricing systems that lack transparency [15].

In the recent past, technological innovation and drug personalisation have spurred the debate on innovative treatments and consequently on the pricing policies to promote them. The value for money of some commercialised drugs is one of the issues. In oncology, for example, ex post data have shown that several active principles have not lived up to their expectations, as the difference between the expected effectiveness and actual survival is large [16], and patient responses have been heterogeneous [17]. This evidence has reopened the debate on the use of risk-sharing arrangements and other forms of price reduction [18]. Another important issue in drug pricing is related to personalisation. The ability to tailor medications to patients produces benefits in terms of health outcomes, but it also has several drawbacks. Effectiveness decreases as soon as the target user is not exactly matched, while development costs and prices are determined to increase. In the context of incomplete information, personalisation may be used as an instrument to avoid competition [19,20,21,22,23] and to increase prices [24,25,26,27,28,29]. On top of this, the pharmaceutical industry enjoys some of the highest returns among the manufacturing sector [30], which are usually not shared equally among countries [31]. The debate on whether healthcare expenditure is sustainable is still prominent in political and economic agendas [25,32], and there does not seem to be a consensus on how to create a more equitable and affordable healthcare system.

In this context, competition, regulation, and pricing mechanisms play a strategic role [33,34,35,36,37,38], even though prices are heterogeneous even among the countries where regulation is in place [39,40,41]. The objective of regulation is to set a price that may allow one to balance value for money (defined as the difference between patient benefit and price) and the profit obtained by the firm. This process should ensure a more equitable allocation of between patient welfare and industry profit, but it may reduce access [42]. If the price is too low (either because of the pricing mechanism or as a result of a risk-sharing agreement), then the industry may decide to not list some indications (leading to the problem of static efficiency [43]), or it may even decide to not develop new active principles (leading to dynamic efficiency problems [43] and a reduction in research). In this article, we review the performances of some of the most widely used price schemes in a framework where drug effectiveness may be uncertain. We show that avoiding a trade-off between access and expenditure is usually almost impossible, but some guidelines can be drawn. First of all, information asymmetry and uncertainty should be taken into account. However, since the level of uncertainty is already quite high, it is advisable for the regulator to set clear rules that the industry may anticipate. Finally, given that the pharmaceutical market is one of the most innovative, regulation should also follow suit by making its process as innovative as possible [44].

## 2. Methods

We present some of the most interesting results from the literature for drug pricing in the context of asymmetric information and patient heterogeneity. To compare the results of the different models proposed in the literature, we set up a simple framework that could be adapted to changes in the characteristics of the drugs or the information set. The common feature of price determination is the presence of a contract between the regulator or payer and industry [43,45,46,47,48]. Models usually differ in their assumptions about the characteristics of the active principle, in the information that the players have at the time of bargaining, and in the type of negotiation chosen (a true bargaining or an all-or-nothing solution). We started from an ideal world, where information is perfect and symmetric. In this framework, it is possible to set an “equitable” price that also allows fair access to an innovative drug. We then moved to a setting where this ideal position could not be reached because of uncertainty and asymmetry of information. The number of patients that may benefit from treatment with a new active principle was normalised to one, but reimbursement could be admitted only for a fraction n∈[0,1] of them. Similar to [45], it was assumed that heterogeneity in effectiveness was due to the following:

- Observable patient characteristics, which determine the “distance” from the profile of the ideal patient for which the drug was developed;
- Individual characteristics that cause heterogeneity in effectiveness within groups of patients with similar characteristics.

Observable patient characteristics capture personalisation. Since drugs are targeted to patients with specific characteristics, their effectiveness depends on how well patients fit with the ideal target. (The effectiveness depends on the disease or different treatment protocols for the same disease, but outcomes may also be determined by patients’ characteristics. For example, the work in [49] shows a case for streptokinase, which, at the time, was the standard of care for the thrombolytic treatment of acute myocardial infarction with tissue plasminogen activator (t-PA). The authors showed that such an active principle had a different effectiveness than expected.

Individual characteristics capture the uncertainty of the effectiveness of a drug among patients of the same type. Randomised clinical trials show the potential benefits of a drug, but true effectiveness may vary due to unpredictable causes, such as unexpected long-term effects or dependence on the active principle. We assumed that observable characteristics allow patients to be clustered into different groups, which can be ordered by increasing distance x from the target. Within each group, effectiveness is a random variable, and we assumed that its average value decreases with increasing distance *x* from the target. Within each group, effectiveness is a random variable and we assumed that its average value decreases with *x*, thus restricting access to fraction *n* of the eligible population within a certain distance from the target, which is equivalent to allowing reimbursement to patients who are likely to benefit most from the treatment. Patients in the target group (x=0) enjoy the highest average effectiveness, which is equal to $\bar{b}$; as patients move away from the target, the average effectiveness decreases steadily and reaches the minimum $\underline{b}$. To simplify the analysis, we assumed that there is a continuum of groups and that the expected marginal effectiveness *M* is a linear function of the fraction *n* of patients with values in the interval $\left[\underline{b},\bar{b}\right]$, which can be written as follows:

(1) $$M\left(n\right)=\bar{b}\;\left(1-n\right)+\underline{b}\;n=\bar{b}-∆b\;n=\frac{\bar{b}+\underline{b}}{2}+∆b\;\left(\frac{1}{2}-n\right),\;∆b=\bar{b}-\underline{b}$$

(for a more general setting of the problem with nonlinear *M*, see [50]). The quantity $∆b=\bar{b}-\underline{b}$ captures the degree of heterogeneity, as it represents the difference between the maximum and minimum expected benefit across groups; as ∆b tends to zero, the marginal benefit tends to a constant value, i.e., the effectiveness does not vary across patient groups. This parameter is also related to the degree of personalisation of the drug: the higher the ∆b, the more personalised the drug.

**Figure 1:**
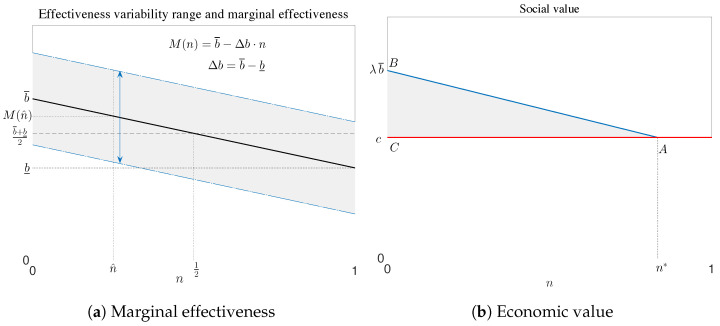
(**a**) The solid line represents the expected marginal effectiveness in Equation (1), while the grey area shows the variance in effectiveness within and between groups. (**b**) Optimal access to the drug from a societal perspective: the grey area represents the expected money equivalent value of the drug, which is measured as the benefit of the drug minus its cost.

Figure 1a shows the two sources of heterogeneity described above. If access is granted to fraction *n* of patients, they belong to a group for whom effectiveness has a range of variation, which is represented by the two dashed-dotted lines, with the solid line describing its expected value (which could be measured, for example, in terms of statistical life years or Quality Adjusted Life Years (QALY)). Because the groups are ordered according to how well their characteristics match the target ailments for which the drug was developed, moving farther to the right reduces the expected effectiveness decreases. While the position of patients on the line can be observed, the variation within each group depends on characteristics that cannot be observed: effectiveness varies in the range between the two arrows, and M(n) is its mean value.

Effectiveness can be translated into a monetary measure by considering that health, along labour, is an input in the productive process. From an economic point of view, healthier individuals contribute with more productively to their activities and increase the value of production. The value to society of treating patients can be determined by multiplying the right-hand side of Equation (1) by λ, the shadow value of health [51,52,53]. The value of the drug to society (which measures the added value of innovation), is given by the difference between the gain in productivity gain from the treatment and its cost. The value for money for the healthcare system depends on the price of the drug, which determines the portion of the gain that is appropriated by the industry in the form of profit. From the societal point of view, if the cost of production is equal to *c*, there may exist a value $n^{*}$ such that the economic value is higher than the cost for all $x<n^{*}$ and is lower for $x>n^{*}$, as shown in Figure 1b. In this case, the maximum economic value is reached when access is restricted to the fraction $n^{*}$ of patients. This value is represented geometrically by the difference between the area under the curve *M* and the rectangle under the horizontal line at height *c*, whose maximum is represented by the area of the triangle ABC.

The (money equivalent) benefits of the new active principle are shared between the industry and consumers through the price set for the new drug. Active principles are usually approved for sale usually by a government agency (for example, the Food and Drug Administration (FDA) in the U.S.), or by a supranational agency (for example, the European Medicine Agency (EMA) in the EU), but are reimbursed only if there is an agreement between the payer and the industry on price. In private and social insurance healthcare systems, prices are set through a negotiation between the insurance/third-party payer and the pharmaceutical company; in public healthcare systems, there is a more formal process, often referred to as the listing process [54].

## 3. Results

Let us first consider an ideal world in which there is no uncertainty and information is symmetric, i.e., both parties know the expected marginal effectiveness of the new active principle as well as the R&D and production costs. This ideal situation is referred to as first best (FB) and is used as a benchmark to evaluate the different pricing schemes. In FB, the industry and the regulator/third-payer negotiate the price, and the drug is reimbursed only to the fraction of patients whose marginal expected marginal benefit is greater than the production cost at a price that shares the benefit of the active principle between the two players [42,45,50,52,55].

**Figure 2:**
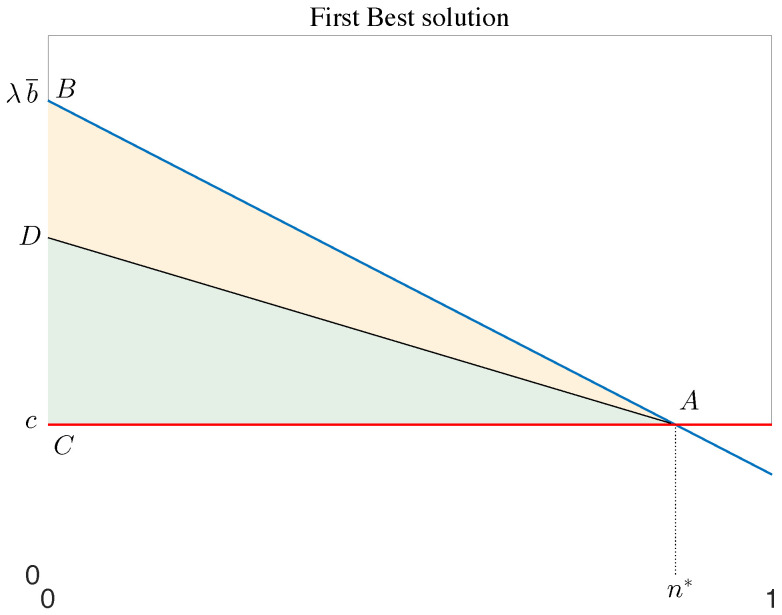
First-best solution. The red line through *C* and *A* denotes the marginal cost *c*, while the marginal expected benefit from using the drug is represented by the blue line through *B* and *A*. Thus, the area of the triangle CAB is the economic value of the drug, which is split by the segment AD into the benefit accruing for society (yellow upper part) and the profit of the industry (green lower part).

Excluding corner solutions (where either no access or universal access is optimal), the situation can be represented as shown in Figure 2: the fraction of patients to be treated is chosen so that the marginal benefit for the “last patient” equals the marginal cost and is denoted by $n^{*}$. The line AD shows how the expected economic value of the drug (the area of the triangle CAB) is divided between consumer surplus (the area of the triangle DAB) and industry profit (the area of the triangle CAD). The price $p^{*}$, which depends on the relative bargaining power of the two actors, determines the slope of AD: the higher the price, the steeper the line, and the lower the value for money for consumers.

The FB solution can be applied to any type of drug, regardless of how effectiveness varies across groups. Note that access is set at the level where marginal benefit equals marginal cost, not price. In other words, when effectiveness is verifiable and common knowledge, heterogeneity in patient responses does not require any innovative or sophisticated model to grant access to the drug at affordable prices.This result is consistent with those of Hlavka et al. [45], who argued that multiple prices are not essential for achieving an optimal allocation even when effectiveness is heterogeneous across patients.

The line AB represents the expected (money equivalent) value of the treatment, not its actual realisation. Ex post, if the actual effectiveness is higher than expected, the drug represents a better value for money for consumers; if lower than expected, value for money decreases. As the price is set at the time of listing, the industry’s profit is not affected by these changes, and the risk associated with uncertainty in the intrinsic effectiveness of the active principle is borne by the payer.

The FB solution is usually not feasible because the market is characterised by uncertainty and asymmetry of information. In addition, without price regulation, the industry could induce consumers to pay a price equal to the maximum allowable price. For these reasons, most healthcare systems use other forms of price-setting mechanisms, ranging from negotiation based on cost effectiveness (C/E) thresholds, value-based pricing, reference price schemes, and managed entry agreements (MEAs). In the following sections, we review their main characteristics.

### 3.1. Second-Best Solutions

In countries where healthcare is funded by the public sector, prices are set through procedures that are not always transparent due to uncertainty and incomplete information. For this reason, bargaining is often replaced by indirect mechanisms that may depend on “all or nothing” agreements or restrictions on the use of new active principles. Cost effectiveness thresholds are one of the first mechanisms used by regulators to reduce uncertainty in the listing process and the welfare losses uncertainty causes [42]. One of the first healthcare systems to use them was the National Institute for Clinical Excellence (NICE), which set an explicit ceiling on reimbursement per unit of effectiveness [56]. In this process, the industry proposes a price, and listing is only granted if the incremental cost effectiveness ratio (ICER) is below a specific threshold. This is usually set either by reference to the extra output a statistical life may produce or by willingness-to-pay considerations [51,52,57].

The introduction of these price caps has stimulated researchers to assess the advantages and disadvantages of such a system [42,58,59,60]. The scheme does have some limitations: unlike in FB, the price is independent of production costs, and it increases the probability that the industry will charge a price very close to $p_{max}$, the price for which the cost/effectiveness threshold is reached [42,58,59]. In the example presented in the previous section, if the threshold is set at λ (which is the maximum), for an average expected effectiveness of *b*, any price below or equal to λb grants listing. As a result, the line AD in Figure 2 rotates clockwise to coincide with AB, i.e., the value of the drug is equal to the industry’s profit.

Moreover, some authors have pointed out that these thresholds may reduce the incentives to invest in innovation [61,62,63], especially for very expensive active principles whose cost may be higher than the price ceiling implicitly set by the C/E threshold.

Several options have been proposed in the literature to increase value for money; the first one we considered is making the listing process uncertain, as in Levaggi [42], where approval depends on the price proposed by the industry. The maximum price is set as before using a C/E threshold, but listing is not granted to any drug that meets this requirement: the probability of being listed depends on the gap between the prosed price and $p_{max}$. This system makes it possible to limit price increases, but it is always inefficient ex post because the payer does not list all the drugs that are potentially cost-effective. Another option, often used by the NICE in the UK, is to restrict access to the new drug to patients who are expected to benefit above a set threshold. In other words, with the evidence produced by the industry, the regulator authorises reimbursement only for a subset of patients, as in the case of pembrolizumab [64]. This mechanism uses the results of a stratified cost-effectiveness analysis (SCEA) to determine the C/E ratio for each subset of patients [49], but only leads to better value for money if the industry does not anticipate this policy. If the industry anticipates this restriction, Hawkins and Scott [65] showed that the manufacturer may propose a combination of price and target patients to be treated that allows increases in profits.

The schemes described above show the fundamental dilemma that regulators face when they do not have the same information as the industry: if they privilege access to the drug, they face a very high price that may make expenditure unsustainable. Moreover, since prices are set on the basis of expected effectiveness, the payer bears all the risk if the drug turns out to be less effective. In some cases, this difference can be quite large, leading to poor value for money. Some authors have recently proposed adjustments to the use of C/E thresholds in price negotiation to reduce price dynamics. For example, Capri et al. [66] proposed setting the price using C/E thresholds, but to reimburse effectiveness that exceeds a specific ceiling. In this way, two important results are achieved: very small improvements in effectiveness (usually survival), which patients may not perceive as significant, are not reimbursed (so that price dynamics are reduced); and, at the same time, incentives for drug research are still quite strong. R&D incentives are indeed a hot topic in the field, as we show below.

### 3.2. Value-Based Price Schemes

This form of payment system was proposed by Gravelle [43] but did not become operational until several years later [53,67,68,69,70]. The rationale behind this is that, unlike other markets where costs can be used to regulate price, for drugs, the starting point should be benefits that patients receive, and the price should reflect effectiveness. With the advent of personalised medicine, the definition of effectiveness also becomes important.

So far, much of the attention has been on marginal value-based prices (MVBPs), where the price is defined as the monetary benefit of the treatment for the marginal patient because of their cost-containment properties, although they may reduce incentives to innovation [22,71]. Average value-based prices (AVBPs), where the price is defined as the monetary benefit of the treatment for the average patient, are more effective in promoting innovation, especially when effectiveness is highly heterogeneous [71], but usually implies a higher price. Finally, when there are large differences in cost effectiveness among patients treated with the same active principle, the literature proposes the use of indication-based prices (IVBPs), where the price may be indication-specific [22,24,25,47,53,72,73,74]. The use of IBP stems from the observation that some active principles used to treat cancer are listed for so many indications [72,75,76,77] that a single price may not be appropriate.

Value-based pricing shifts the risk of high production costs to the industry: two active principles with the same effectiveness are priced the same, but the industry’s profits are inversely related to the marginal cost. However, it is important to remember that the idea behind value-based schemes and cost/effectiveness thresholds is precisely to relate the price to the intrinsic value of the drug (in terms of health improvement) rather than its production costs. The mainstream literature [5,53,67,68,70,78,79] suggests using a marginal value-based price (MVBP) to allow for a fairer price. If the price is equal to the willingness of pay of the group of patients with the lowest benefits, for the others, the price is lower than the benefit enjoyed. However, some important aspects are not taken into account: (a) in the listing process, the payer cannot force the industry to increase the number of groups for which listing is requested if the latter decides to restrict them in order to keep price high, and (b) the pharmaceutical company may have better information than the payer about the effectiveness of the new drug.

If this is the case, Levaggi and Pertile [71] showed that under MVBPs, access is restricted by the strategic listing of the industry: in other words, there is once again a trade-off between prices and access to the drug. The authors showed that AVBPs may represent better value for money: they make it possible to treat the same number of patients as in FB but at a price that allows part of the rent to be distributed to consumers. To understand this point, let us look again at Figure 2. Let us first assume that the payer can observe both *c* and the line AB. The marginal value-based price would be *c*, and the total economic value of the drug would be consumer surplus. This is the argument in the literature supporting this view. However, both *c* and the knowledge of *M* may be private information for the industry, which could decide to partially reveal it to obtain a higher price. In this case, the profit of the industry is positive, and the number of people receiving the drug is inefficiently low.

Let us now turn to an environment where information is asymmetric. Levaggi and Pertile [80] considered the case where a monopolist wants to maximise profit in a static framework. The information about heterogeneity across patients is private to the firm; in other words, with reference to Equation (1), the payer can only observe $M\left(\frac{1}{2}\right)$, i.e., the average expected effectiveness. The industry can reveal the true ∆b, or it can declare that ∆b=0, i.e., that the effectiveness is not heterogeneous across patients. In this context, it is shown that all the value-based formulas produce a similar result: under IVBPs and AVBPs, the industry is indifferent to revealing heterogeneity across patients; under MVBP, by not revealing such information, they obtain a price that is equal to the average effectiveness. In all cases, the value of the drug in is fully appropriated by the industry. An insight can be gained by looking at Figure 1a. Suppose that the true marginal benefit is represented by the solid line, and it is private information of the firm. Under MVBP, declaring that expected effectiveness is constant (the dashed line) is profitable: the firm receives the same payment for each patient.This behaviour may not be ethical: if the industry has some information, it should share it with the payer; it is important to note here that under a price scheme such as MVBP, the industry has no incentives to invest into researching the effectiveness differential.

In a dynamic context where a firm is a monopolist in a period but will compete with another firm in the future, Levaggi and Levaggi [81] analysed the incentives to reveal (and research) the effectiveness differential across payment systems. They showed that personalisation is indeed used to differentiate the product from that of the competitor. For example, this could be the strategy pursued by the producer of pembrolizumab to enter the market for oncological drugs without direct competition with nivolumab [82]; a similar strategy might have been put forth by the producers keytruda, an immuno-oncological drug [21]. It also turns out that personalisation is the optimal strategy for the entrant rather than the incumbent, a result in line with the empirical evidence [75]. There may also be a trade-off between access and personalisation. For example, with AVBP, the first firm that enters the market has no incentive for drug personalisation, so the listing grants access to all the patients; on the contrary, the MVBP creates an incentive to personalise but to list only for the most effective indications. Brekke et al. [21] compared the results of a monopoly case where only one industry sells a drug in a market where two industries compete. They showed that even in this case, industries can use personalisation to reduce competition and induce the purchasers to also include drugs in their formulary that are less effective. In other words, personalisation can also be used to commercialise lower-quality drugs.

## 4. Drug Price When Effectiveness Is Uncertain

Most of the schemes presented so far explicitly address only with the first dimension of heterogeneity in patient responses (observable heterogeneity) but leave all the consequences of uncertainty in ex post effectiveness to the payer. Hlavka et al. [45] showed that indication-based pricing can partially solve the problem of uncertainty in patient outcomes, but this often requires a level of information that may not be available to the payer. In fact, IBPs may only be implemented if patients are stratified into groups, with additional costs for clinical trials [20]. For some drugs, in the early stages of phase III of their development, the only verifiable information may be an average measure of outcome [83], but the industry may still observe differences across patients groups. Such information can be valuable to both the patients and the industry: the industry may charge a higher price, and patients may be better off, but only if information is correct. More research into the effectiveness of the active principle may lead to verifiable information but with a significant delay in the listing process. An alternative option could be the use of performance-based, anaged entry agreements (PBAs): these schemes introduce a form of risk sharing, whereas the industry agrees to pay back part of the price if the ex post effectiveness falls below a specific threshold. The introduction of risk sharing has sparked a lively debate in the literature about its desirability [79,84,85,86,87,88,89,90,91]. Its use in combination with value-based prices and MEAs integrates several components we presented previously: a C/E threshold that defines a ceiling on the price, or a base price, that can be proposed by the industry or set by the regulator, which is a rebate that depends on ex post effectiveness. These agreements are compared in [50] with indication value-based price schemes. In a framework with asymmetry of information, IBPs allow optimal access only at the cost of handing over all the social value of the drug as profit to the industry, while PBAs can be effective if risk sharing is not too aggressive, and prices are set outside the negotiation process. In other words, these schemes allows drugs to be listed earlier, but rebates do not allow expenditure to be reduced substantially [25,92].

## 5. Discussion

The trade-off between access (i.e., being able to treat all patients for whom the drug is effective) and value for money (negotiating a price that allows some of the benefits of the active principle to accrue to the healthcare system) is at the heart of price regulation. In an ideal world, these objectives could be achieved simultaneously, but, in the real world, several factors can undermine this possibility. For this reason, healthcare regulators use a wide range of policies, often in combination, resulting in a system that is not transparent and, in any case, not efficient in optimising both access and innovation in the long run [14,15].

In a context of information asymmetry, the industry can reduce the value for money of innovative drugs through strategic listing. Payers can try to limit expenditure growth, but this usually means restricting access. C/E thresholds do not seem to be effective in reducing prices, while risk-sharing agreements increase uncertainty for both the industry and patients [93,94].

Value-based pricing, which has recently been proposed to overcome some of the problems of indirect pricing mechanisms such as C/E ratios, does not seem to live to its expectations: when information asymmetry is taken into account, the industry may strategically exploit heterogeneity in patient responses to capture the main share of the value of innovation, as shown above. In the recent past, PBAs have been proposed as a solution to balancing early access to care with value for money. They are still less common than financial MEAs, but their number is growing rapidly [55,95,96,97,98,99].

Assessing the impact of PBAs empirically is difficult because rebates are usually not disclosed. From a theoretical point of view, it has been shown that risk sharing should be rather soft, and prices should be set outside the MEA. These results suggest that these agreements are more suitable for making innovations available before their effectiveness has been fully verified than for containing prices.

In the recent past, some regulators (e.g., in Germany) have also used external reference pricing rules, where the threshold is determined on the basis of the (weighted) average price in other countries.

Houy and Jelovac [38,100] showed that their application may delay listing in some countries, while Bardey et al. [33] pointed out that they may reduce expenditure in R&D. Finally, Voehler et al. [101] showed that the price itself does not seem to change dramatically as a result of this regulation. The issue of investment in innovation is certainly very important as pricing may change the timing of the launch of new technologies and the return on investment in innovative drugs [100,102,103,104,105,106,107]. It should be noted, however, that researchers [108] recently argued that a fair share of the value of the drug to leave the industry would be about 20%, while, in general, this is much higher; in other words, the incentives to invest into innovation seem to still be quite high.

## 6. Conclusions

Nearly one-fifth of total healthcare expenditure in OECD countries is spent on the purchase of drugs, so the rise in their price can lead to a (possibly) unsustainable burden, especially for publicly funded healthcare systems. The pharmaceutical market is changing: active principles are increasingly personalised, which may improve effectiveness but also increase the cost of innovation. In this context, price regulation is essential. Price dynamics should be regulated with the dual objective of achieving a fair distribution of the economic value of innovation (static efficiency), while maintaining incentives to research new treatments (dynamic efficiency). In this article, we examined the results in terms of access (the number of patients benefiting from the introduction of new active principles) and affordability (expenditure for providers) of some pricing schemes proposed to reimburse new active principles. Two sources of volatility in effectiveness were taken into account: an intrinsic element and a patient-dependent element. The results are quite interesting. If we simply take into account the heterogeneity of patient responses across groups, i.e., there is no uncertainty in the effectiveness once patients have been sorted by some observable elements, value-based pricing is a fairly effective instrument for setting price. However, we showed that once information asymmetry is taken into account, there does not seem to be much difference in the allocation of the value of the drug across pricing schemes. Value-based schemes are not effective in improving value for money for consumers. When uncertainty is also taken into account, the picture becomes more clouded. Some value-based schemes such as IBPs allow an efficient number of patients to be treated but usually at the cost of allocating the full economic value of the drug to the industry in the form of profits. On the other hand, schemes such as PBAs may allow a fairer distribution, but, in this case, the payer should be careful in setting the thresholds for paying the rebate. This review of the models proposed in this article showed that information asymmetry is at the heart of this problem and should be recognised by payers in the architecture of pricing schemes and that more research is needed to find mechanisms that can balance equity and efficiency considerations.

## Data Availability

No new data were created or analyzed in this study.
